# Success in Africa: People with disabilities share their stories

**DOI:** 10.4102/ajod.v8i0.522

**Published:** 2019-04-25

**Authors:** Tom Shakespeare, Anthony Mugeere, Emily Nyariki, Joseph Simbaya

**Affiliations:** 1 Norwich Medical School, University of East Anglia, Norwich, United Kingdom; 2 Department of Sociology and Anthropology, Makerere University, Kampala, Uganda; 3 School of Public Health, University of Nairobi, Nairobi, Kenya; 4 Institute of Economic and Social Research, University of Zambia, Lusaka, Zambia

**Keywords:** disability, education, employment, Africa, achievement

## Abstract

**Background:**

Whereas most narratives of disability in sub-Saharan Africa stress barriers and exclusion, Africans with disabilities appear to show resilience and some appear to achieve success. In order to promote inclusion in development efforts, there is a need to challenge narratives of failure.

**Objectives:**

To gather life histories of people with disabilities in three sub-Saharan African countries (Kenya, Uganda and Sierra Leone) who have achieved economic success in their lives and to analyse factors that explain how this success has been achieved.

**Methods:**

Qualitative research study of economic success involving life history interviews with 105 participants with disabilities from both urban and rural settings recruited through disabled people’s organisations and non-governmental organisation partners, framework analysis of transcripts to chart success and success factors.

**Results:**

Participants had faced barriers in education, employment and family life. They had largely surmounted these barriers to achieve success on an equal basis with others. They were working in private and public sectors and were self-employed farmers, shopkeepers and craftspeople.

**Conclusion:**

The findings of this study suggest that, given the right support, disabled people can achieve economic success, with the implication being that investment in education or training of disabled people can be productive and should be part of overall development efforts for economic reasons, not solely to achieve social justice goals.

## Background

In understanding the experience of disability in Africa, particularly in terms of poverty and livelihoods, there appears to be a paradox. Disabled Africans face structural factors such as barriers in the environment, in education and in employment, as well as widespread discriminatory attitudes, that limit their chances of achieving success on an equal basis with others (Banda-Chalwe, Nitz & De Jonge 2013; Groce et al. 2011; WHO 2011). For example, Eide and Ingstad (2013) summarise the findings of the 2002–2013 series of seven SINTEF surveys on disability in Africa:

Key indicators on education, mental and physical health, employment, socio-economic status, access to information, social participation, et cetera all point in the same direction: there are substantial gaps in services to disabled people, disability is associated with a lower level of living when compared to non-disabled persons, women with disabilities are worse off than males, and the rural disabled have a lower level of living than their urban counterparts. (p. 2)

The SINTEF data are confirmed by other studies across the African continent (Trani & Loeb 2012; Trani et al. 2010). International data confirm that this situation is general for people with disabilities in low- and middle-income settings. From Filmer (2008), it is evident that adults with disabilities are more likely to live in poorer households. Mitra, Posarac and Vick (2011) analyse multidimensional poverty among people with disabilities in low- and middle-income countries. Persons with disabilities are disproportionately unemployed or economically inactive. A detailed study of access barriers for people with mobility impairment in Zambia shows some of the reasons why people with disabilities are so often excluded from education, employment, transport and other social goods (Banda-Chalwe et al. 2013). Similarly, there are barriers to participation for deaf people, as was found in Uganda (Mugeere et al. 2015).

As a consequence of this exclusion from public life, and the worlds of education and employment, at first glance prevailing cultural representations of disability in Africa appear overwhelmingly negative, featuring discourse of people with disabilities as ‘economically invalid and economically dependent’ (Tsemma 2014:145). Even in Uganda, negative attitudes are common, despite the vigour of the disability rights movement (Abimanyi-Ochom & Mannan 2014). As in most developing countries, a person with disability is more likely to be seen begging at the traffic lights than to be encountered as a teacher or doctor or shopkeeper (Groce et al. 2013). Faced with these barriers to achievement, it is no surprise that research finds deep cultural beliefs about disability and incapacity across the African continent (Munsaka & Charnley 2013; Swartz & Marchetti-Mercer 2017).

Yet at the same time, anyone who spends time working with people with disabilities in Africa – or many other low-income settings – has encountered dozens of successful, assertive, proud persons with disabilities. Often, these folk are associated with vibrant disabled people’s organisations (DPOs), but increasingly they have moved on into mainstream roles in society. In reviewing qualitative studies, Eide and Ingstad (2013) point to the importance of capturing the agency of people with disabilities, while balancing that with an awareness of the structural forces that make it so difficult for people with disabilities and their households to survive, let alone thrive. Hearing the voices of people with disabilities living in poor conditions is emphasised: they suggest that while disability and poverty are linked, some people with disabilities do manage:

Individuals with disabilities living in poverty do struggle to survive and to make the best out of their situation – and there are encouraging examples of individuals who have used their disability as a resource for themselves and for others in the community. (Eide & Ingstad 2013:6)

The danger could be that the achievement of these more successful persons with disabilities remains largely invisible, which could mean that investment in disabled people by national governments or international donors – whether in education, vocational rehabilitation, employment, or social protection – might be considered to be a waste. Funding participation of people with disabilities might thus be considered to be more of a charitable or humanitarian response, rather than a development priority. Yet this thinking risks excluding 15% of the population (WHO 2011). This is not simply a moral problem; it could also be an economic mistake: for example, Buckup (2009) argued, in a study for International Labour Organisation which included Zambia, that exclusion of people with disabilities from the world of work causes economic losses of 3.7% of GDP.

Kenya, Uganda and Zambia have all ratified the Convention on the Rights of Persons with Disabilities, as well as domestic civil rights legislation. For example, in 2012, Zambia passed the *Persons with Disabilities Act No. 6*, aimed at promoting equal participation by persons with disabilities. In Uganda, the 2006 *Persons with Disabilities Act* offers tax credits on employers who take on 10 or more persons with disabilities, as well as obliging workplaces to make modifications, a similar approach to Kenya’s 2003 *Persons with Disabilities Act* (Tsemma 2014). There is still, however, a long way to go before everyone understands disability to be a human rights issue, not a charitable issue (Abimanyi-Ochom & Mannan 2014; Tsemma 2014); there is an implementation gap between the positive legislative picture and the situation on the ground (Abimanyi-Ochom & Mannan 2014; Owens & Torrance 2016).

World Bank data, where available, casts light on the situation in these three countries. Kenya, Uganda and Zambia are comparable on health indicators. Life expectancy is reported to be 61.3 for Zambia and 59.5 for Uganda. The contrasting role of government health services is indicated by data on out-of-pocket health expenditure, which comprises 26.1% of healthcare costs in Kenya, 30% of healthcare costs in Zambia and 41% of healthcare costs in Uganda. Of note is the fact that 11% of Zambian children and 6.2% of Ugandan children are out of school. Zambia, thanks to copper exports, has the highest GDP per capita at $1627, followed by Kenya at $1143 and Uganda at $662. In each setting, most people live in rural areas and a minority live in urban areas (2013: 25% of Kenyans, 15% of Ugandans and 40% Zambians).

The complexity of trying to understand individual disability successes within the backdrop of disadvantage fuelled the Department for International Development/Economic and Social Research Council-funded research project reported in this paper, which had the aim of exploring factors that explained the success of some persons with disabilities on the African continent. Success was not firmly defined in advance by the researchers, but was defined locally. Success was predominantly understood in economic terms. To be successful was to enjoy economic prosperity on an equal basis with others, to use the language of the UN Convention on the Rights of Persons with Disabilities. In this project, the researchers were looking for persons with disabilities who were either working as self-employed people or employed in mainstream, DPO or non-governmental organisation (NGO) settings. Very few of them could be classified as wealthy, but all of them were getting by, and most of them were able to have their own home and start a family.

In order to home in on this criterion of economic independence, for the purposes of this study people with intellectual disabilities were excluded because they were considered to be less likely to have achieved this goal, although it was recognised that some people with intellectual disabilities were contributing to their households. People with mental health conditions were also excluded because these are often fluctuating situations, with complex impacts on livelihood. In limiting the study to people with physical and sensory impairments, the researchers did not negate the difficulties of these other population groups, nor the fact that some of them could be considered successful on the same or different terms. They aspired to study these experiences in a future project. The current paper reports on the findings about the extent and nature of success experienced by these participants, whereas a subsequent paper will analyse the factors that might explain this success.

## Methodology

This study was conducted in Kenya, Sierra Leone, Uganda and Zambia, although data from Sierra Leone are not reported in this paper. The aim is to complete transcription and publish the Sierra Leone data in a subsequent publication.

This study entailed conducting in-depth life story interviews with persons with disabilities in both urban and rural settings who had experienced success in their lives. The research team did not attempt to define what success was precisely, although the recruitment material was explicit about economic success being the main criterion. The researchers did not seek those who were remarkable – for example, Paralympians or millionaires – but instead sought out everyday life stories of individuals who had succeeded on an equal basis with others. The ambition was to recruit approximately equal numbers of men and women.

Recruitment was via DPOs, such as National Union of Disabled Persons of Uganda, Association of Disabled Persons of Kenya; as well as through NGOs, such as Leonard Cheshire Disability; and also, government agencies such as Zambia Agency for Persons with Disabilities. Efforts were made to recruit half of the participants in urban and half in rural settings. In Uganda and Zambia, participants were purposefully recruited from different regions of the country, as well as the capital cities, using existing networks. The Kenyan team also recruited from both Nairobi and a rural district. It was not at all difficult to recruit people with disabilities who were perceived to be successful in their communities, drawing on existing NGO and DPO networks. However, although the team set out to conduct 40 interviews in each setting, the final data set comprised only 104 participants, which was mainly because of constraints on staff time. There are a further 12 transcripts from Sierra Leone, although 40 interviews have been conducted there.

All authors were responsible for the interviews. All interviews were recorded and then transcribed. Analysis was conducted using the Framework Analysis approach (Ritchie & Spencer 1994). After reading and rereading the transcripts, a framework was constructed according to the themes which emerged from the stories. A separate framework was created for each country, using Excel. A separate section of the framework was created to cover individual, family education, employment, attitudes and help received. The next stage was charting, where each transcript was analysed according to the framework, and summaries were added to the Excel spreadsheet. For each country, at least three different coders created, added or revised the framework, reaching a consensus as to how to analyse the data most completely and accurately. Use of the Framework Analysis approach, based on Excel spreadsheets, made it easy to compare participants and also to share the data analysis across the three African countries as well as UK and South Africa.

### Ethical considerations

Information about the study was provided to all participants, each of whom gave written or in a few cases oral consent. All data were anonymised after transcription. Ethics review was conducted by the Ethics Committee of the university and local research ethics committees and permissions were granted. This study received ethical clearance from the Ethics Committee of University College London (1661/007), as well as from relevant ethics committees in Kenya (NACOSTI/P/16/92785/12347), Uganda (SS4207) and Zambia (2016-Mar-011).

## Findings

### Impairment

This was an opportunity sample, and the breakdown of respondents reflects the local activities of Leonard Cheshire Disability and the relevant DPOs who distributed the request for participants, as well as the personal networks of the researchers and other individuals involved in the Bridging the Gap Research programme (Table 1).

**TABLE 1:** Impairments of participants in study.

Variable	Kenya	Uganda	Zambia
Hearing loss or deaf	2	5	0
Sight loss or blind	4	9	7
Albino	2	2	2
Restricted growth	0	3	2
Polio survivor	10	10	11
Other mobility impairment	11	9	12
Not otherwise specified or missing	2	3	0
Women	17	16	6
Men	14	24	28
**Total**	**31**	**40**	**34**

In general, the distribution of impairments among the participants was similar across the three settings. The majority of impairments were either congenital – such as albinism or restricted growth – or else resulted from illness or trauma which occurred before the age of 18. The impact of polio was disaggregated from the other mobility impairments because it was so dominant, although it should be noted that polio is no longer endemic to these three countries. In general, it was interesting to note that the majority of the impairments were preventable: for example, blindness resulting from measles, cerebral palsy resulting from malaria, paralysis resulting from polio or a road traffic injury, and impairments resulting from violence. Perhaps because the intention was to sample persons with disabilities of working age, there was no one who was primarily affected by the typical late-onset conditions which are familiar from high-income settings, such as diabetes, or stroke, although there was one individual with arthritis.

### Education

Between a tenth and a third of participants had failed to complete school, mainly owing to barriers. With these exceptions, the remaining participants were well educated, whether they had attended special schools or mainstream schools. While they came from diverse socio-economic backgrounds, the participants generally appeared to have very good intelligence and emotional intelligence, meaning that they were better able to benefit from their education and overcome the obstacles they faced, whether around travel to school or in the classroom itself. It should be noted that according to the psychological literature, intelligence has been found to be one of the key factors underlying resilience. Because they showed individual promise, several participants were successful in attracting interest from relatives or benefactors who were willing to pay school or university fees. Those who had been lucky enough to reach university often had their fees paid by the government (Table 2).

**TABLE 2:** Highest educational level of study participants.

Stage achieved	Kenya	Uganda	Zambia
Did not complete school	10	4	7
Secondary	2	3	8
Diploma	8	8	8
Graduate	4	16	4
Postgraduate	5	8	5
Not otherwise specified or missing	3	1	2
**Total**	**31**	**40**	**34**

Those who did not complete secondary schooling were more likely to be in rural areas, where factors like lack of money to pay school fees explain the lack of formal qualifications. Some individuals who did not complete school had gone on to undertake vocational rehabilitation courses, for example, as a cobbler or as a knitter. Individuals attaining diplomas tended to have undergone teacher training or attended agricultural college or become accountants. Uganda in particular had astounding educational achievements, with nearly two-thirds of participants achieving university degrees, twice the success of Kenya and Zambia. Two Ugandans had studied at Kenyan Universities, one at a Netherlands university. One Kenyan participant had gone to university in Rome, another had studied in Canada.

This evident success masks the barriers that participants had overcome in order to complete their education. For example, a Kenyan woman with mobility impairment had been unable to attend a school which was 3 km away, until her grandfather made her walking sticks and so from age 10 she could manage the journey. She reported that ‘you had to be tough’ in the face of an unfriendly educational environment’ (Respondent 301). Another Kenyan (Respondent 326) reported losing 8 years of schooling after becoming disabled. Those who attended school (e.g. Respondent 318) faced neglect, problems in accessing the toilet and in having to queue for long periods. But the biggest challenge was the attitudes of others who might mock the disabled person (response 322) or ostracise them (Respondent 312) or bully them, such as the mobility-impaired Ugandan child whose school peers used to take away his walking stick ‘to test him on how he would walk without them’ (Respondent 205). One Ugandan woman with restricted growth reported that teachers as well as pupils would mock her (Respondent 206). Some deaf participants reported being so frustrated at the communication and information barriers they faced that they dropped out of school for periods of time (e.g. Respondent 214). Others had changed schools, sometimes more than once, so as to avoid difficulties.

Where they faced barriers, these participants used ingenuity to overcome those barriers. For example, one Kenyan deaf woman described how she had attended mainstream school. When asked how she coped in a big class, where she could not hear and there was no sign language interpreter, she explained that she copied the notes of the cleverest pupil. Where she did not understand the notes, she looked at another schoolmate’s notes, until she had learned the lesson. In a rote-learning system, she was successful because she worked hard and memorised the notes, and thus passed her examinations. She went on to attend teacher training, and then obtained an education degree and finally postgraduate education. These achievements are more remarkable when it is known that she came from an impoverished background – her parents were uneducated agricultural workers – and faced prejudice – neighbours had told her father that she would never amount to anything and that her deafness was the result of witchcraft. She reported being the only member of her family to have finished school, let alone having tertiary education, and she was now supporting the education of her siblings.

Similar stories were heard from many participants. A regular refrain was the proud participant who said, ‘I was the only member of my family to finish school’, such as the Zambian who told me: ‘It’s like each time I reach a certain stage, I realize I can still do something else’ (Respondent 104). It was common for participants to be sponsoring the education of other siblings or younger relatives. These achievements highlight the comparative success of these disabled respondents when compared with non-disabled family members and the impact of their success on their entire families.

### Employment

In this study, individuals were specifically recruited on the basis of their economic success, so it is not surprising that almost all were employed in the public or private sector or earning a living as farmers or traders. Many individuals who reported, for example, working for a DPO as their main job, also mentioned that they supplemented their salary by subsistence farming, or via commercial activities such as owning and hiring out motorcycles, or tailoring or trading (Table 3).

**TABLE 3:** Livelihood outcomes for participants.

Sector	Kenya	Uganda	Zambia
Civil service employee	5	6	4
Professions (teacher, lawyer, etc.)	2	2	8
NGO or DPO employee	7	14	1
Business employee	1	5	3
Self-employed (retail or trader)	6	5	6
Self-employed (crafts)	5	2	6
Farming	3	2	3
Other or NOS	1	2	1
Unemployed, retired or student	1	2	2
**Total**	**31**	**40**	**34**

DPO, disabled people’s organisation; NGO, non-governmental organisation; NOS, not otherwise specified.

People had had to adapt to different and changing opportunities: for example, the man with visual impairment who had started out as a teacher in a mainstream school, then worked at a special school, then ran a Braille press, then worked for a church organisation. As has been noted, in Uganda, DPOs are very strong. Disability rights issues have a high profile, and disabled people have reserved places at all levels of government: for example, several individuals reported their employment as ‘politician’. Conversely, DPOs appear weaker in Zambia. This may be why very able people with disabilities in Zambia were working as professionals – teachers, accountants etc. – whereas in Uganda these very able people might be working for DPOs.

Participants discussed various barriers in mainstream employment. Some had faced discrimination from employers or from co-workers: for example, this was mentioned by half of the Kenyan participants. This had either barred access to mainstream employment entirely or hindered further promotion for those who had been successful in getting jobs. People tried hard to be independent, for example the Kenyan participant who strived to cope for work, not asking for help ‘unless it is very necessary’ (Respondent 333). The negative attitudes towards people with disabilities also affected some participants who needed capital, such as the Ugandan woman who sought a loan from a microfinance institution and was asked ‘but will you manage to pay us back our money?’ (Respondent 229).

A common response to limited employment options was to resort to farming or other self-employment. Some respondents worked in the garment industry – as tailors, embroiderers, knitters or cobblers. In rural areas, respondents kept pigs, bees, or chickens or had a fish farm or produced fruit or peanut butter. For those who had not even completed school, there were few other options – although one Ugandan (Respondent 234) had taught himself photography and made a living as a photographer, supplemented by farming.

For example, one Zambian participant (Respondent 108) had experienced a T4 spinal cord injury while at college, completing most of his education from hospital. He was not aware of any relevant DPO. However, as he said, ‘OK this is just a disability, I can still use my hands and I can still use my brains, let me see what I can do’. Facing discrimination in the open job market, he saved money to start his own business. He ran a grocery shop (retail and wholesale) and employed two people. Furthermore, he was sponsoring the education of his late sister’s child and another unrelated child.

Ugandans working in the DPO sector often mentioned they had taken this career path after facing discrimination in mainstream roles, for example, the legal assistant who co-founded a DPO in response to her limited employment options (Respondent 241). However, a number of other participants stated they had not experienced discrimination or that they had been well supported in the workplace.

Above all, the clear thread that runs through the stories of people who were disabled in childhood is the vital importance of education (Lamichhane & Okubo 2014). For those who become impaired as adults, the key is to ensure they can gain relevant vocational skills for the local market and where necessary achieve access to the unconditional cash transfers (Handa et al. 2018) or microfinance loans which are required to set up a business or in farming or to improve productivity of these economic strategies. Again, participants who lacked education were sometimes disadvantaged when they developed disability in midlife and could no longer work the land and had no or few other economic options (e.g. Respondent 232).

### Family life

The birth family of these participants could be both a hindrance and a support. For example, many participants reported negative attitudes or ignorance among relatives. For example, a birth mother left because she could not cope with her son’s needs (Respondent 320); a grandmother initially encouraged the parents to leave the child to die (Respondent 319); a father walked out on the family because he thought educating the disabled child was a waste of time (Respondent 318). Similarly, an older brother ‘does not accept or respect me’ (Respondent 312), or in other cases (e.g. Respondent 311) children went away to boarding school to gain an education, but then struggled to reintegrate into their birth family.

Yet this was not the whole story. Many other participants highlighted the love and support of the mother, father, uncle, aunt, brother, sister or grandparent who enabled them to believe in themselves and fight for inclusion. Sometimes the support was very practical, such as the young man whose brothers would carry him 5 km to get to school, and then carry him between classes and to the toilet (Respondent 234). Other participants told us ‘I cannot even begin to think of how my life would have been without family support’ (Respondent 305) or ‘My parents love me, and they show the others that I am a child to them just like the others’ (Respondent 330). The mother who told her blind son ‘You, of all my children, must finish school’ echoed the importance with which education was regarded by many parents of disabled children.

True acceptance for many disabled people means not just getting an education and finding a job, so as to achieve self-sufficiency, but also finding a partner and having a family. Of the 31 Kenyan participants, 23 were married or in long-term relationships, and in all, they had 54 biological children. Of the 39 Ugandan participants, 22 were married or in long-term relationships, with a total of 84 biological children from this cohort. Of the 34 Zambian participants, 26 were married or widowed, with a total of 110 biological children. In addition, it was a very common theme to hear participants proudly mention that they were supporting their siblings through education or they were looking after their elderly parents. Forming a family also added to economic security, for example through having another, often non-disabled, person to assist in farming and other livelihood activities, as well as in household and parenting tasks, and having children or siblings who grow up as economically active family members able to support the disabled individual in old age.

People did face difficulties in having a family. Several men and women reported that partners left them after they developed a disability (e.g. Respondent 204, 223, 228). Others had to overcome the prejudices of their partners’ families (e.g. Respondent 299). Although their economic success could make men eligible as partners, despite disability, this seemed less possible for women. For example, a Kenyan woman who made a living as a hawker blamed the prejudice of her mother-in-law for her divorce. A Kenyan participant (Respondent 302) with phocomelia (missing arms) had gained a Bachelor’s degree and then a Master’s degree from the UK and had subsequently flourished in the civil service. She said: ‘You do what you can, at least to change the mind of those negative ones, and there are also positive ones who come to embrace you.’ She was now looking forward to retiring to a property with land that she had invested in. Although impairment had curtailed romantic relationships, this had enabled her to focus on her career. After all, as she reported: ‘for a disabled person … sometimes having those relationships, they make you more sad.’ At the time of interview, the Zambian participant (Respondent 108) whose economic success is reported above remained unmarried, lived alone and reported that he was keen to start a relationship.

This theme of gender inequalities was taken up by a Ugandan woman with restricted growth who told us that she was determined to avoid men. She had other women friends with restricted growth who had been befriended by non-disabled men, who would visit them in the night for sex and who then leave them when they became pregnant. The same story was also told to us by people with albinism and other visible impairments: it appeared that certain unscrupulous non-disabled men were only interested in experimenting with sex with ‘exotic’ women but had no intentions of marrying them. As one woman from Uganda reported, non-disabled men ‘use and discard’ women with disabilities (Respondent 225).

## Discussion and conclusions

The intention of this paper was to report on the evidence of success among some people with disabilities in Africa. The data shared here challenge the negative assumptions that imply people with disabilities can never do well, or always need hand-outs. The participants in this study had become extremely self-directed, resilient and positive individuals, who were contributing not just to the well-being of their families but also to the economic development of their society. For example, the Kenyan disabled man (Respondent 314) who said: ‘I want to show my fellow PWDs that we can make it, and when I fully recover I have a vision that I want to create job opportunities for others’. Begging was not the only option for persons with disabilities (Groce et al. 2013).

In the absence of government support, these individuals were overcoming obstacles and making progress on their own. Another participant said: ‘If I start pitying myself I will fail, and nobody is caring about me and nobody is willing to help me, so I have to cope with whatever comes ahead of me’. This highlighted how, in the absence of a welfare state or other supports, many individuals with disabilities in developing countries have no choice but to rely on their own resources if they want to survive, let alone thrive.

This study adds to our knowledge about persons with disabilities in Kenya, Uganda and Zambia, joining other grassroots studies but expanding our understanding of the circumstances in which people with disabilities can achieve success on an equal basis to others. Grassroots qualitative studies add richness to the picture of disability in sub-Saharan Africa.

This qualitative study was not representative of all persons with disabilities. To follow up these findings, a larger quantitative study, representative of adults with disabilities, could adopt a case–control methodology to understand the differences between those individuals who achieve success and those who are unable to overcome barriers. It is urgently important to understand what factors make the difference, and where the levers of success are amenable to government or donor interventions. Moreover, this study only included people with sensory and physical impairments. It would be important to conduct specific studies of people with mental health conditions and people with intellectual disabilities who are flourishing, in order to reveal what factors account for their success.

There are risks associated with reporting these success stories. For example, the stereotype of failing Africans with disabilities could be replaced by contrasting stereotype of ‘super crip’ courageous Africans with disabilities overcoming adversity. A reaction could also be: if these individuals are succeeding thanks to their own efforts, why cannot other individuals with disabilities do likewise? This is why it is important to understand success not just in terms of individual resilience but also in terms of structural factors that enable individuals to achieve. Rather than shallow stereotypes, offering the full range of complex stories of real individuals counters this one-dimensional representation.

The stories shared here suggest that investing in people with disabilities, and in barrier removal efforts in education, employment and the wider environment makes good sense, in terms of economic development. For example, the Ugandan government policy of financing university enrolment of students with disabilities appears to have increased the likelihood of Ugandans with disabilities having degrees, as compared to persons with disabilities from neighbouring countries. Just as other evidence from the non-disabled population shows individuals and families succeeding in escaping poverty (Krishna et al. 2006; Lawson, McKay & Okidi 2006). It was also evident from these data that some determined people with disabilities in Kenya, Uganda and Zambia were succeeding, against considerable odds, in enjoying success on an equal basis with others. These success stories may still be minority experiences, but they can contribute to challenging the negativity and prejudice that surrounds disability in Africa, and indeed other developing countries. The goal of governments and other development actors should now be to implement interventions that address the many barriers that prevent more people with disabilities achieving success.
